# Ultrasonography of the spleen, liver, gallbladder, caudal vena cava and portal vein in healthy calves from birth to 104 days of age

**DOI:** 10.1186/1751-0147-55-68

**Published:** 2013-09-16

**Authors:** Ueli Braun, Sonka Krüger

**Affiliations:** 1 Department of Farm Animals, Vetsuisse Faculty, University of Zurich, Zurich, Switzerland

## Abstract

**Background:**

Many of the ultrasonographic abdominal findings of adult cattle probably also apply to calves. However, significant changes associated with ruminal growth and transition from a milk to a roughage diet occur in young calves during the first few months, and it can be expected that ultrasonographic features of organs adjacent to the rumen such as spleen and liver also undergo significant changes. These have not been investigated to date and therefore the goal of this study was to describe ultrasonographic findings of the spleen, liver, gallbladder, caudal vena cava and portal vein in six healthy calves from birth to 104 days of age. Standing calves were examined ultrasonographically six times at three-week intervals starting on the first or second day of life using a 5.0-MHz transducer and techniques described previously.

**Results:**

The spleen was imaged on the left at the 5th to 12th intercostal spaces. The dorsal and ventral visible limits ran from cranioventral to caudodorsal because of superimposition of the lungs. The size of the spleen was largest at the 7th and 8th intercostal spaces and the maximum thickness was measured at the 9th to 12th intercostal spaces. The liver was seen in all calves on the right and could be imaged at the 5th to 12th intercostal spaces and the area caudal to the last rib. Similar to the spleen, the dorsal visible margin of the liver ran parallel to the ventral border of the lungs. The visible size of the liver was largest at the 8th to 11th intercostal spaces and the maximum thickness was measured at the 8th and 9th intercostal spaces. The parenchymal pattern consisted of numerous fine echoes homogeneously distributed over the entire organ. The gallbladder was most commonly seen at the 9th intercostal space and was circular, oval or pear-shaped on ultrasonograms. It extended beyond the ventral border of the liver depending on the amount of bile. The caudal vena cava was triangular in cross section but sometimes had a round or oval profile and was always seen in at least one intercostal space. The maximum circumference was measured at the 10th and 11th intercostal spaces. The portal vein was circular or oval in cross section and was characterised by stellate ramifications branching into the liver parenchyma. The portal vein could always be imaged at the 7th to 11th intercostal spaces and its mean diameter at the 9th to 11th intercostal spaces ranged from 1.2 cm to 1.8 cm.

**Conclusions:**

The ultrasonographic findings of the spleen, liver, gallbladder, caudal vena cava and portal vein in six healthy Holstein-Friesian calves from birth to 104 days of age serve as reference values for the examination of these anatomical structures in diseased calves.

## Background

The ultrasonographic findings of the spleen, liver, gallbladder, caudal vena cava and portal vein of adult cattle have been described in detail [1-3]. These organs have also been characterised ultrasonographically in goats [4,5] and, with the exception of the spleen, in sheep [6]. In calves, similar reports are only available for newborns [7]. Umbilical disease is conceivably the most important indication for ultrasonography in calves [8-10]; in calves with omphalophlebitis, the enlarged umbilical vein can easily be followed ultrasonographically from the naval to the liver. Imaging umbilical abscesses is also straightforward [8,10]. The spleen and liver may also be the site of disease processes in calves. Neoplasia is not uncommon and may result in severe lesions in one or both organs [11-14]. The spleen may be involved in septicaemic processes associated with omphalitis or bronchopneumonia [15], and hepatitis and liver abscess are common seqelae of navel infection [16]. Although rare, portosystemic shunt is another anomaly of calves; in one affected calf, the connection between the cranial mesenteric vein and the caudal vena cava could be visualised [17]. In another calf with portosystemic shunt, the lesions found at necropsy were characterized by increased numbers of arteriolar profiles and hypoplasia to absence of portal veins [18]. In a calf with abdominal situs inversus, two speens could be detected at necropsy [19]. Many of the ultrasonographic abdominal findings of adult cattle probably also apply to calves. However, significant changes associated with ruminal growth and transition from a milk to a roughage diet occur in young calves during the first few months, and it can be expected that ultrasonographic features of organs adjacent to the rumen such as spleen and liver also undergo significant changes. These have not been investigated to date and therefore the goal of this study was to describe ultrasonographic findings of the spleen, liver, gallbladder, caudal vena cava and portal vein in six healthy calves from birth to 104 days of age.

## Methods

The study protocol was approved by the Animal Care Committe of the Canton of Zurich, Switzerland.

### Animals

Six clinically healthy Holstein Friesian bull calves were enrolled in this study within one day of birth. The results of clinical, haematological and biochemical examinations were within normal ranges and have been published elsewhere [20]. The calves were bovine-virus-diarrhoea virus antigen negative.

### Study design

The spleen, liver, gallbladder, caudal vena cava and portal vein of each calf were examined six times at three-week intervals (Table 1) and 3.0 to 5.5 hours after the calves had been fed cow’s milk. The calves were weaned after examination 4 and then fed hay ad libitum. In addition, the calves received calf starter (Ufa-Aufzuchtfutter, UFA AG, Zollikofen) and a small amount of haylage.

**Table 1:** Age of calves, interval between ingestion of milk and examination and amount of milk fed (mean ± sd, range in brackets) at six ultrasonographic examinations during the first 104 days of life

**Examination**	**Age (days)**	**Hours after feeding**	**Amount of milk fed (litres)**
1	1.9 ± 1.14	4.6 ± 0.57	1.7 ± 0.50
	(1.0 – 4.0)	(3.0 – 5.5)	(1.0 – 3.0)
2	19.7 ± 1.79	4.7 ± 0.43	2.2 ± 0.27
	(16.0 – 23.0)	(4.0 – 5.5)	(2.0 – 3.0)
3	41.0 ± 0.76	4.9 ± 0.38	2.6 ± 0.42
	(40.0 – 43.0)	(3.5 – 5.5)	(2.0 – 3.0)
4	62.1 ± 1.08	4.7 ± 0.60	2.6 ± 0.44
	(60.0 – 64.0)	(3.0 – 5.5)	(2.0 – 4.0)
5	82.8 ± 1.03	Weaned	Weaned
	(81.0 – 85.0)		
6	99.2 ± 3.08	Weaned	Weaned
	(95.0 – 104)		

### Ultrasound machine

A real-time B-mode ultrasound machine (EUB 8500, Hitachi Medical Systems, Zug) and a linear 5.0-MHz transducer (Type EUP L53) were used.

### Preparation of calves and ultrasonographic examination

The calves were clipped on both sides from behind the shoulder to the caudal flank and from the transverse processes of the thoracic and lumbar vertebrae to the ventral midline. The skin was cleaned with alcohol and lubricant (Vetogel®, Streuli Pharma AG, Uznach) was applied. A contact gel (Aquasonic®, Polymed, Opfikon/Glattbrugg) was also applied to the transducer. The calves stood during the examination and were not sedated.

### Ultrasonography of the spleen

Each intercostal space on the left was scanned, beginning dorsally and progressing ventrally with the transducer held parallel to the ribs. The spleen was first assessed subjectively by evaluating the visibility of the capsule and the ultrasonographic pattern of the parenchyma. The position of the dorsal and ventral visible limits of the spleen were assessed in each intercostal space to determine the size and location of the spleen analogous to the technique used in adult cattle [2] and goats [4]. The position of the dorsal and ventral visible limits of the spleen were determined in relation to the midline of the back using a measuring tape, and the visible size of the spleen was obtained by subtracting the distance between the dorsal limit and the midline from the distance between the ventral limit and the midline. In each intercostal space the maximum splenic thickness was measured on ultrasonograms using the electronic cursors.

### Ultrasonography of the liver

The liver was examined on the right side from the 5th intercostal space to the region caudal to the last rib in a dorsal-to-ventral direction with the transducer held parallel to the ribs using a technique described for adult cattle [1], sheep [6] and goats [5]. The liver parenchyma was first assessed subjectively by determining the appearance of the surface, the echogenicity and pattern of the parenchyma and whether the hepatic blood vessels could be visualised. The size of the liver was calculated in each intercostal space analogous to the method used for the spleen [2] and as described for adult cattle [1] by using the distances between the dorsal and ventral visible limits of the liver and the dorsal midline. The thickness of the liver was measured in each intercostal space at the level of the portal vein using the electronic cursors while the image was frozen.

### Ultrasonography of the gallbladder

The intercostal spaces in which the gallbladder could be seen were first determined, and then the shape, size, content and appearance of the wall of the organ were assessed. The maximum length and width were measured on ultrasonograms using the electronic cursors.

### Ultrasonography of the caudal vena cava and portal vein

The intercostal spaces at which these blood vessels could be visualised and the appearance of the vessels were recorded. The distance from each blood vessel to the diaphragmatic surface of the liver, the circumference of the caudal vena cava and the diameter of the portal vein were measured on ultrasonograms using the electronic cursors.

### Statistical analysis

A statistical software program (STATA 12, StataCorp LP, Collage Station, Texas, USA, 2011) was used to calculate means, standard deviations and frequency distributions, and the Wilk-Shapiro test was used to test data for normal distribution.

## Results

### Ultrasonographic findings of the spleen

**Figure 1:**
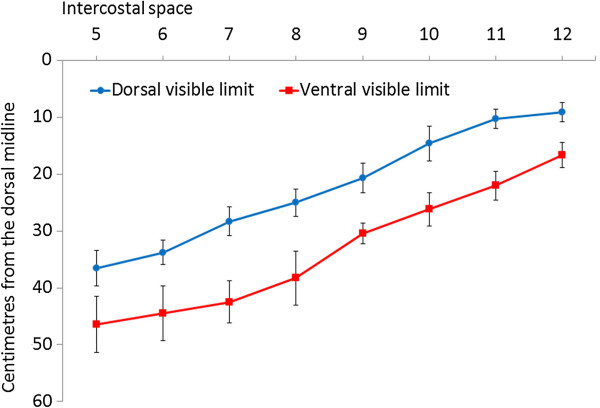
**Dorsal and ventral visible margins of the spleen.** Dorsal and ventral visible margins of the spleen at the 5th to 12th intercostal spaces in six healthy Holstein-Friesian calves at examination 3 (approximate age, 6 weeks; mean ± standard deviation).

**Figure 2:**
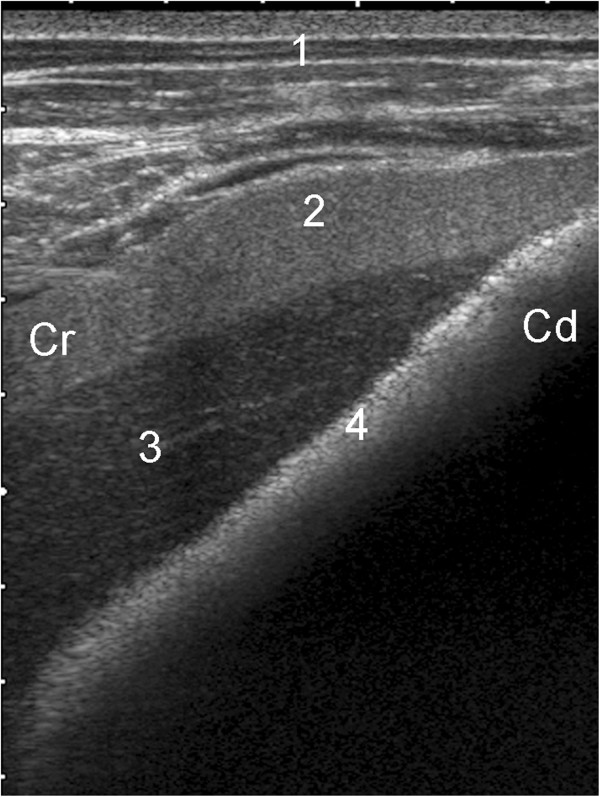
**Ultrasonogram of the spleen and liver.** Ultrasonogram of the spleen and liver in a 61-day-old Holstein-Friesian calf viewed from the ventral midline. 1 Ventral abdominal wall, 2 Spleen, 3 Liver, 4 Reticulum, Cr Cranial, Cd Caudal. (depth of the image = 8 cm).

**Figure 3:**
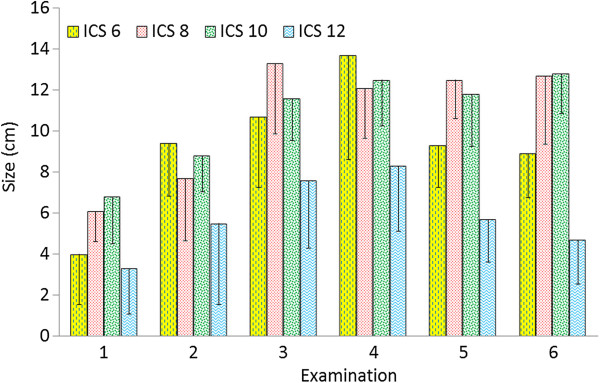
**Size of the spleen.** Size of the spleen (distance between the dorsal limit of the spleen and the midline of the back subtracted from the distance between the ventral limit of the spleen and the midline) at the 6th, 8th, 10th and 12th intercostal spaces in six healthy Holstein-Friesian calves at examinations 1 to 6 (days 1.9 ± 1.14 [examination 1], 19.7 ± 1.79 [examination 2], 41.0 ± 0.76 [examination 3], 62.1 ± 1.08 [examination 4], 82.8 ± 1.03 [examination 5], 99.2 ± 3.08 [examination 6]) (mean ± standard deviation). ICS Intercostal space.

**Figure 4:**
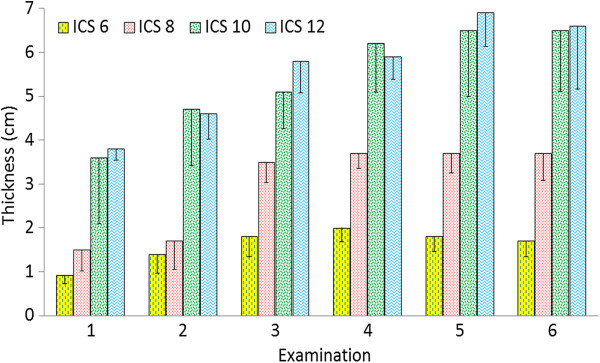
**Thickness of the spleen.** Thickness of the spleen at the 6th, 8th, 10th and 12th intercostal spaces in six healthy Holstein-Friesian calves at examinations 1 to 6 (see Figure 3) (mean ± standard deviation). ICS Intercostal space.

The spleen could be visualised in all calves on the left side. It was situated between the costal part of the abdominal wall and the rumen or the reticulum. The dorsal part of the spleen was in contact with the diaphragm and superimposed by the lung. The spleen could always be seen at the 8th to 11th intercostal spaces, but the range of visibility varied at the remaining intercostal spaces. The dorsal and ventral visible margins of the spleen ran from cranioventral to caudodorsal because of superimposition of the lung (Figure 1), and the distances between these margins and the midline of the back were therefore the largest in the 5th and the shortest in the 12th intercostal space. At examinations 2 to 4, the spleen extended ventrally far enough to contact the liver in five calves (Figure 2). The size of the spleen in the intercostal spaces increased from examinations 1 to 4, after which there was little change (Table 2). The size was the largest at the 7th and 8th intercostal spaces and the smallest at the 5th and 12th intercostal spaces (Figures 1 and 3). The thickness of the spleen also increased from examinations 1 to 4 (Figure 4, Table 2); the smallest and largest measurements were obtained at the 6th to 8th and 9th to 12th intercostal spaces, respectively. The parenchymal pattern of the spleen consisted of numerous fine echoes homogeneously distributed over the entire area of the organ. The splenic vessels appeared in the parenchyma as elongated hypoechoic structures in longitudinal section and circular to oval hypoechoic structures in cross section. The capsule of the spleen was imaged as a fine hyperechoic line on the diaphragmatic surface, but not on the visceral surface because of superimposition of the rumen.

**Table 2:** Size and thickness of the spleen and the liver, circumference of the caudal vena cava, and diameter of the portal vein in calves from birth to 104 days of age (mean ± sd, range in brackets, all measurements in cm)

**Organ (location)**		**Examination**					
	**Variable**	**1**	**2**	**3**	**4**	**5**	**6**
Spleen (ICS 7)	Size	5.4 ± 1.9 (3.5 – 8.5)	8.8 ± 3.5 (4.5 – 13.5)	14.2 ± 2.1 (11.0 – 16.0)	14.3 ± 3.5 (10.0 – 19.5)	13.7 ± 3.5 (9.5 – 18.0)	10.9 ± 4.1 (7.0 – 17.0)
	Thickness	1.1± 0.4 (0.6 – 1.7)	1.9 ± 0.7 (1.1 – 2.9)	2.5 ± 0.6 (1.9 – 3.4)	2.7 ± 0.7 (2.0 – 3.6)	2.6 ± 0.7 (1.6 – 3.4)	2.5 ± 0.5 (1.8 – 3.2)
Liver (ICS 9)	Size	14.8 ± 3.8 (10.0 – 20.0)	15.6 ± 3.2 (10.5 – 18.5)	17.8 ± 6.2 (11.0 – 28.0)	19.5 ± 3.7 (14.0 – 25.0)	11.2 ± 0.6 (9.5 – 13.5)	12.4 ± 2.5 (9.0 – 15.5)
	Thickness	5.8 ± 0.6 (5.1 – 6.9)	6.4 ± 0.4 (5.8 – 7.1)	7.2 ± 0.8 (6.2 – 8.1)	7.6 ± 0.8 (6.3 – 8.9)	7.6 ± 0.9 (6.2 – 8.8)	8.0 ± 0.7 (6.2 – 7.5)
Caudal vena cava (ICS 10)	Circum-ference	4.2 ± 0.7 (3.4 – 4.9)	5.3 ± 0.7 (4.5 – 6.0)	6.5 ± 1.0 (5.6 – 8.5)	6.2 ± 1.1 (4.9 – 7.7)	5.5 ± 0.9 (4.2 – 6.6)	5.6 ± 1.4 (3.8 – 7.8)
Portal vein (ICS 10)	Diameter	1.4 ± 0.2 (1.1 – 1.6)	1.7 ± 0.2 (1.3 – 1.9)	2.0 ± 0.3 (1.5 – 2.4)	1.9 ± 0.3 (1.5 – 2.3)	1.8 ± 0.3 (1.5 – 2.1)	1.8 ± 0.3 (1.4 – 2.2)

*ICS* Intercostal space.

### Ultrasonographic findings of the liver

**Figure 5:**
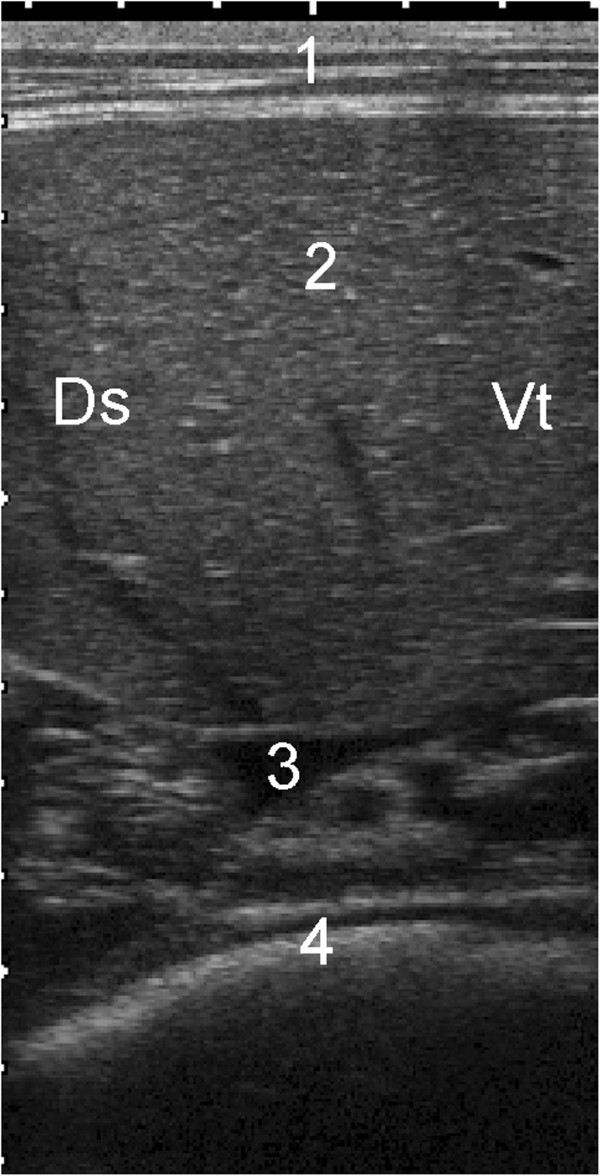
**Ultrasonogram of the liver and caudal vena cava.** Ultrasonogram of the liver and caudal vena cava viewed from the 11th intercostal space on the right in a 95-day-old Holstein-Friesian calf. 1 Abdominal wall, 2 Liver, 3 Caudal vena cava, 4 Rumen, Ds Dorsal, Vt Ventral. (depth of the image = 12 cm).

**Figure 6:**
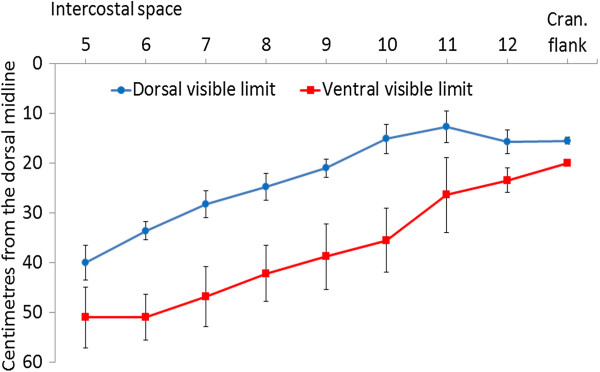
**Dorsal and ventral visible margins of the liver.** Dorsal and ventral visible margins of the liver at the 5th to 12th intercostal spaces and the cranial flank on the right side in six Holstein-Friesian calves at examination 3 (41.0 ± 0.76 days old) (mean ± standard deviation).

**Figure 7:**
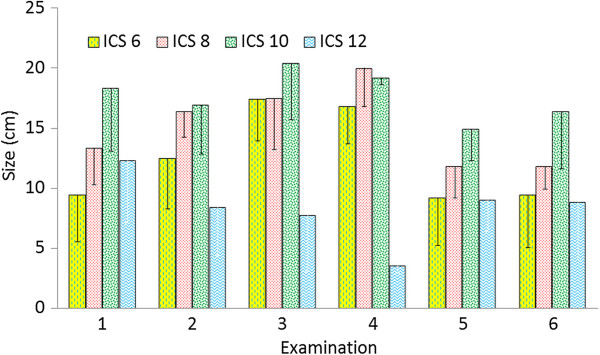
**Size of the liver.** Size of the liver (distance between the dorsal limit of the liver and the midline of the back subtracted from the distance between the ventral limit of the liver and the midline) at the 5th to 12th intercostal spaces in six healthy Holstein-Friesian calves at examinations 1 to 6 (see Figure 3) (mean ± standard deviation). ICS Intercostal space.

**Figure 8:**
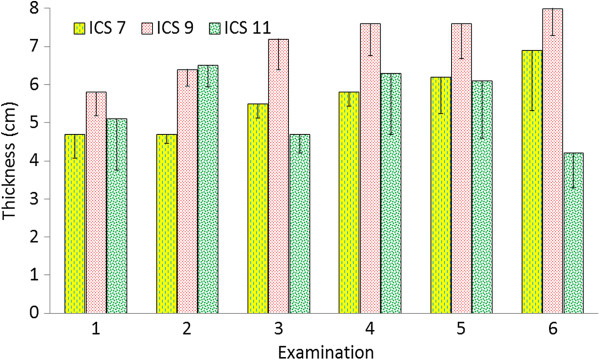
**Thickness of the liver.** Thickness of the liver at the 7th, 9th and 12th intercostal spaces in six healthy Holstein-Friesian calves at examinations 1 to 6 (see Figure 3) (mean ± standard deviation). ICS Intercostal space.

The parenchymal pattern of the liver consisted of numerous fine echoes homogeneously distributed over the entire area of the organ (Figure 5). The diaphragmatic surface was imaged in all calves as a fine hyperechoic line adjacent to the peritoneum, but the visceral surface was not always clearly defined. The contour of the liver at the distal angle was generally rounded. The branches of the portal vein and the hepatic veins imaged in the parenchyma increased in size toward the portal vein and caudal vena cava, respectively. The wall of the portal vein was generally better defined than that of the hepatic veins because of an echogenic rim, but clear differentiation was only possible in the area where stellar ramifications of the portal vein branched into the parenchyma. The intrahepatic bile ducts could not be seen. The liver was always seen at the 5th to 12th intercostal spaces and the area behind the last rib on the right. Cranially it was adjacent to the diaphragm and dorsally it was superimposed by the lungs as far back as the 11th or 12th intercostal space. The dorsal visible margin of the liver ran parallel to the ventral border of the lungs from cranioventral to caudodorsal (Figure 6). The distance of the dorsal visible margin of the liver from the dorsal midline decreased caudally because the liver became less obscured by the lung. The distance between the dorsal visible margin of the liver and the dorsal midline was greatest at the 5th intercostal space and shortest at the 11th intercostal space. The ventral margin of the liver had a similar course; it was furthest from the dorsal midline at the 5th intercostal space and closest to the dorsal midline at the cranial flank. The visible size of the liver was largest at the 8th to 11th intercostal spaces and was considerably smaller cranially and caudally (Figures 6 and 7). The liver did not increase in size noticeably from examination 1 to 4, but the visible size at the intercostal spaces was smaller at examinations 5 and 6 than at examinations 1 to 4 (Table 2). The thickness of the liver increased between examinations 1 and 3 and changed little thereafter (Figure 8, Table 2). The maximum thickness was measured at the 8th and 9th intercostal spaces.

### Ultrasonographic findings of the gallbladder

**Figure 9:**
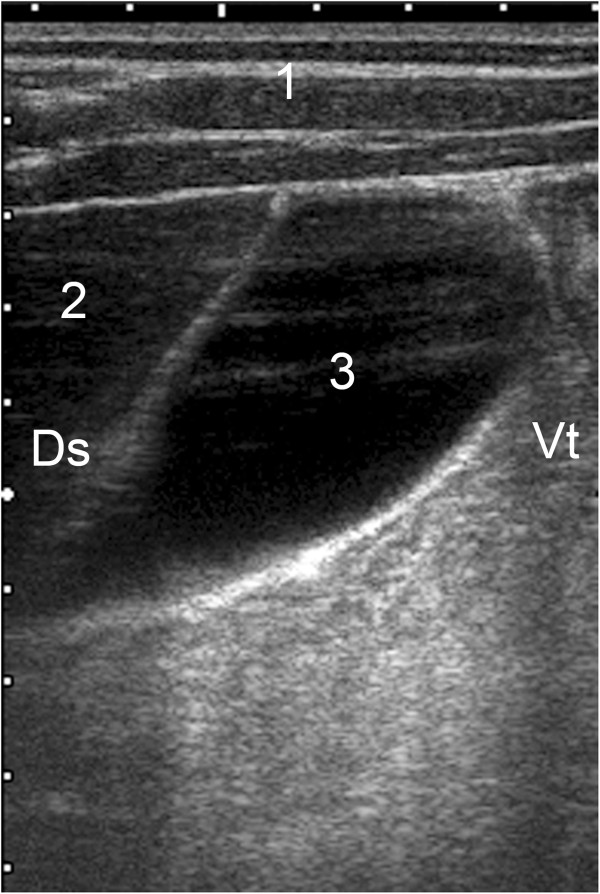
**Ultrasonogram of the gallbladder.** Ultrasonogram of the gallbladder viewed from the right in a 62-day-old Holstein-Friesian calf. 1 Abdominal wall, 2 Liver, 3 Gallbladder, Ds Dorsal, Vt Ventral. (depth of the image = 9 cm).

The gallbladder was circular, oval or pear-shaped (Figure 9) and sometimes extended beyond the ventral margin of the liver depending on the amount of bile. The content was always hypoechoic and surrounded by an echoic wall. The gallbladder was seen at examinations 1 to 6 in 5, 5, 4, 5, 4 and 6 calves, respectively; it was seen at more than one intercostal space nine times, at two intercostal spaces eight times and at three intercostal spaces once. It was seen most commonly at the 9th intercostal space. The length varied from 1.5 ± 1.27 cm to 5.6 ± 0.00 cm and the width varied from 0.90 ± 0.27 cm to 1.8 ± 0.00 cm.

### Ultrasonographic findings of the caudal vena cava

**Figure 10:**
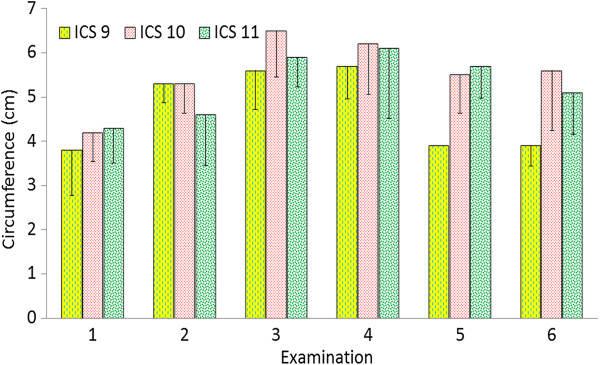
**Circumference of the caudal vena cava.** Circumference of the caudal vena cava at the 9th to 11th intercostal spaces on the right in six healthy Holstein-Friesian calves at examinations 1 to 6 (see Figure 3) (mean ± standard deviation). ICS Intercostal space.

The caudal vena cava was triangular in cross section because it was embedded in the sulcus of the vena cava of the liver (Figure 5); in the calf 2, it was rounded or oval in the examinations 1 and 4, in the calf 3 in the examinations 2, 3, 5 and 6, and in the calf 4 in the examinations 2 and 4. It was always seen in at least one intercostal space. It was seen at the 9th to 12th intercostal spaces at examinations 1 and 2 and at the 9th to 11th intercostal spaces at the later examinations. It was not seen at the 5th to 8th intercostal spaces because of superimposition of the lungs. The distance between the caudal vena cava and the dorsal midline varied from 13.2 ± 1.30 cm to 21.8 ± 1.06 cm and the distance between the caudal vena cava and the diaphragmatic surface of the liver from 4.8 ± 0.02 cm to 7.7 ± 0.41 cm. The mean circumference of the vessel was largest at the 10th and 11th intercostal spaces and at examination 1 varied from 3.8 cm to 4.3 cm (Figure 10). The circumference increased during the study period; it was largest at 6.5 cm at examination 3 (Table 2).

### Ultrasonographic findings of the portal vein

**Figure 11:**
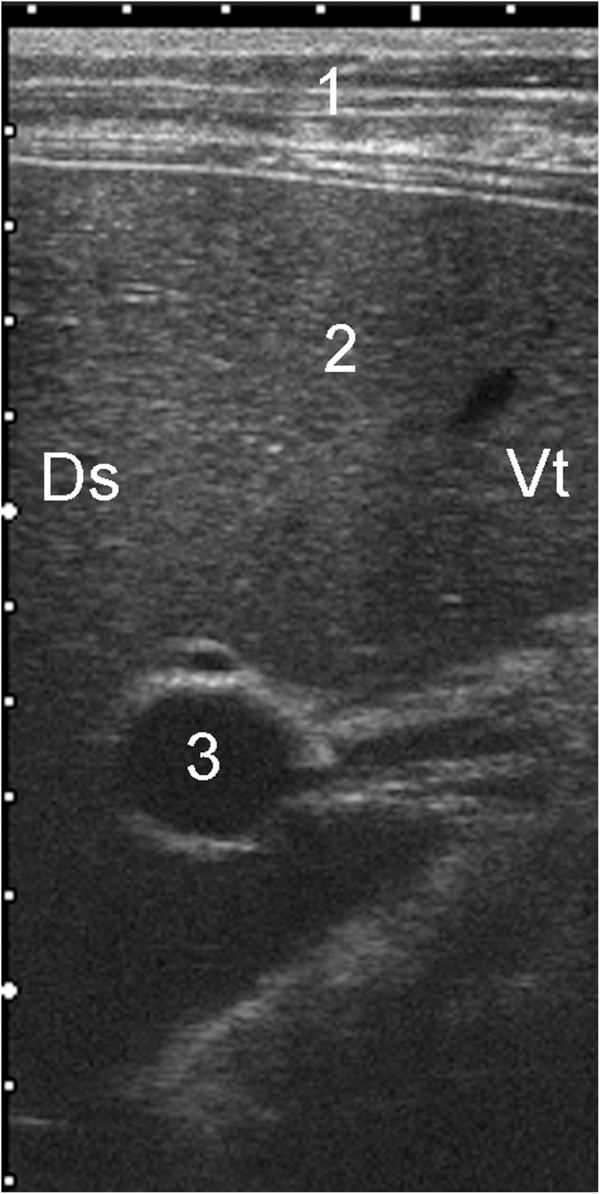
**Ultrasonogram of the portal vein.** Ultrasonogram of the portal vein viewed from the right in an 83-day old Holstein-Friesian calf. 1 Abdominal wall, 2 Liver parenchyma, 3 Portal vein, Ds Dorsal, Vt Ventral. (depth of the image = 12 cm).

**Figure 12:**
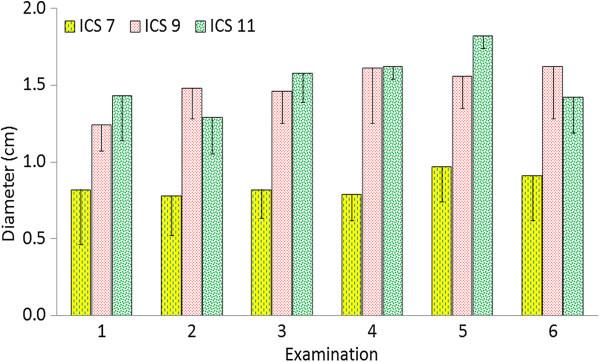
**Diameter of the portal vein.** Diameter of the portal vein at the 7th, 9th and 11th intercostal spaces in six healthy Holstein-Friesian calves at examinations 1 to 6 (see Figure 3) (mean ± standard deviation). ICS Intercostal space.

The portal vein was circular or oval in cross section and had stellate ramifications branching into the liver parenchyma. The wall was more echogenic than the wall of the caudal vena cava and was distinct within the parenchyma of the liver (Figure 11). The portal vein was seen at more intercostal spaces than the caudal vena cava because of a more ventral position and less superimposition of the lungs. It was always seen at the 7th to 11th intercostal spaces and sometimes also at the 6th and 12th intercostal spaces. Compared with the caudal vena cava, the portal vein was always more ventral and closer to the diaphragmatic surface of the liver. The distance between the portal vein and the dorsal midline decreased from cranial to caudal, and the diameter of the vein varied little during the study period (Figure 12). The mean diameter of the vein was smaller at the 7th and 8th intercostal spaces than at the 9th to 11th intercostal spaces where it ranged from 1.2 cm to 1.8 cm (Table 2). At examination 1 the maximum distance measured between portal vein and diaphragmatic surface of the liver was 4.2, and at examination 6, this distance was 5.3 cm.

## Discussion

The ultrasonographic examination of the spleen was straightforward because of its location adjacent to the costal part of the abdominal wall. It was always visible at the 8th to 11th intercostal spaces and sometimes at the 5th to 7th and 12th intercostal spaces. The dorsal and ventral visible margins of the spleen ran from cranioventral to caudodorsal. In contrast to goats, in which the spleen has a dorsal position [4], the spleen extends further ventrally in calves and adult cows and contacts the reticulum. This predisposes the spleen to injury from foreign bodies that penetrate the reticular wall, potentially causing purulent splenitis and splenic abscess [21]. However, compared with adult cattle, hardware disease rarely occurs in calves. The ultrasonographic pattern of the splenic parenchyma was similar to the pattern described in adult cattle [2] and goats [4]. The maximum thickness of the spleen was measured in each intercostal space because, unlike the portal vein in the liver, there is no landmark that allows measurement of the thickness at a defined location. The thickness increased from cranial to caudal.

In adult cattle, reference values for the ultrasonographic examination of the liver were established many years ago [1,22]. The techniques described were also well suited for the examination of young calves in the present study, in which the liver could always be visualised at the 6th to 11th intercostal spaces. The liver can be imaged most frequently at the 10th to 12th intercostal spaces in adult cows [1], at the 7th to 9th intercostal spaces in goats [5] and at the 9th and 10th intercostal spaces in sheep [6]. The difference between species with regard to visibility of the liver at the last two intercostal spaces is most likely related to an anatomical difference between cattle and small ruminants; in the latter, the liver has a more upright position than in the former [23] and therefore is not usually imaged at the last two intercostal spaces. The liver reached across the median into the left hemiabdomen similar to reports in newborn calves [7,24,25].

The caudal vena cava could always be imaged in at least one of the 9th to 12th intercostal spaces, but was not visible cranial to the 9th intercostal space because of superimposition of the lungs. The vein was imaged ultrasonographically in 96% of adult cattle [1]. In healthy adult cattle, sheep and goats, the cross section of the caudal vena cava always has a triangular shape, whereas in calves it can be slightly rounded or oval. In adult ruminants, the vein is triangular because it is embedded in the sulcus of the vena cava of the liver [3]. A circular or oval cross section is not normal and indicates congestion of the vessel, caused mostly by thrombosis or sometimes by right cardiac insufficiency or compression of the vein in the thorax [26,27]. It appears that in calves a slightly rounded or oval cross section of the caudal vena cava is not abnormal. Presumably the caudal vena cava is circular in cross section initially and then changes to a triangular shape because of pressure from the surrounding abdominal organs after they have fully developed. In the young calf, some of the adjacent organs may not be fully developed and the abdominal pressure therefore reduced.

The portal vein was always imaged in at least two intercostal spaces including the 9th and 10th intercostal spaces, and similar to reports in adult cattle [1], was characterised within the parenchyma by a distinct echoic wall. The cross-sectional shape of the vein and the stellate ramifications were similar to those described in adult cattle, sheep [6] and goats [5].

Visibility of the gallbladder was limited to a single intercostal space in most calves. Similarly, in adult cattle and goats, the gallbladder is usually seen in only a single intercostal space [1,5], and in newborn calves it was most often seen in the 9th or 10th intercostal space [7]. While the gallbladder was visualised in only about 20% of calves in the latter study, we could image the gallbladder in 66 to 100% of calves at each examination. The size of the gallbladder varied considerable in this study, which was in agreement with findings in adult ruminants. The emptying reflex of the gallbladder in response to eating or rumination is responsible for frequent changes in size [28]. This also explains why the gallbladder cannot always be visualised ultrasonographically. The shape varied from round to oval as described in cows. The gallbladder was situated medial to the liver or extended beyond the ventral margin of the liver and was then adjacent to the abdominal wall depending on the amount of bile.

## Conclusions

The ultrasonographic findings of the spleen, liver, gallbladder, caudal vena cava and portal vein in six healthy Holstein-Friesian calves from birth to 104 days of age serve as reference values for the examination of these anatomical structures in diseased calves.

## Competing interests

The authors declare that they have no competing interests.

## Authors’ contributions

UB initiated and planned the study and he prepared the manuscript. SK carried out the ultrasonographic examinations under supervision of UB. Both authors have read and approved the manuscript.
